# A Review of Abdominal Meshes for Hernia Repair—Current Status and Emerging Solutions

**DOI:** 10.3390/ma16227124

**Published:** 2023-11-10

**Authors:** Alfred Najm, Adelina-Gabriela Niculescu, Bogdan Severus Gaspar, Alexandru Mihai Grumezescu, Mircea Beuran

**Affiliations:** 1 Department of Surgery, Carol Davila University of Medicine and Pharmacy, 8 Eroii Sanitari, Sector 5, 050474 Bucharest, Romania; alfred.najm@yahoo.ro (A.N.); bogdangaspar2005@yahoo.com (B.S.G.); drmirceabeuran@yahoo.com (M.B.); 2 Emergency Hospital Floreasca Bucharest, 8 Calea Floresca, Sector 1, 014461 Bucharest, Romania; 3 Research Institute of the University of Bucharest—ICUB, University of Bucharest, 050657 Bucharest, Romania; adelina.niculescu@upb.ro; 4 Department of Science and Engineering of Oxide Materials and Nanomaterials, Politehnica University of Bucharest, 011061 Bucharest, Romania; 5 Academy of Romanian Scientists, Ilfov No. 3, 050044 Bucharest, Romania

**Keywords:** hernia repair, abdominal meshes, medical textiles, polymeric composites, emerging solutions, advanced materials, clinical trials

## Abstract

Abdominal hernias are common issues in the clinical setting, burdening millions of patients worldwide. Associated with pain, decreased quality of life, and severe potential complications, abdominal wall hernias should be treated as soon as possible. Whether an open repair or laparoscopic surgical approach is tackled, mesh reinforcement is generally required to ensure a durable hernia repair. Over the years, numerous mesh products have been made available on the market and in clinical settings, yet each of the currently used meshes presents certain limitations that reflect on treatment outcomes. Thus, mesh development is still ongoing, and emerging solutions have reached various testing stages. In this regard, this paper aims to establish an up-to-date framework on abdominal meshes, briefly overviewing currently available solutions for hernia repair and discussing in detail the most recent advances in the field. Particularly, there are presented the developments in lightweight materials, meshes with improved attachment, antimicrobial fabrics, composite and hybrid textiles, and performant mesh designs, followed by a systematic review of recently completed clinical trials.

## 1. Introduction

The human abdominal wall comprises a complex, multilayered structure, assuming the overlapping of skin, subcutaneous fat tissues, several muscle layers, preperitoneal fascia, and peritoneum. These anatomic formations attach to each other and the bone to contain and protect intra-abdominal contents, assure postural assistance, and maintain abdominal pressure. However, when defective areas appear in the abdominal wall, they favor the protrusion of abdominal structures, generally termed “hernias” [1,2,3]. 

Abdominal wall hernias are frequent clinical problems associated with significant rates of disability and morbidity [4]. More than 20 million hernias are operated per annum globally [5], this medical condition being the third most common and liable abdominal pathology and the second pathology of consultation in general surgery in patients of age limits [2]. Despite its prevalence, there is still a lack of knowledge on this condition among patients, who often discover hernias in the late stages of development. Nonetheless, hernias should be repaired at the earliest convenience to avoid severe complications [6,7].

Repairing the herniated abdominal wall represents a challenging task, generally requiring surgical intervention [6,8]. Many developments have been accomplished in hernia repair over the past 60 years, yet there is no single gold standard for the effective management of abdominal wall hernias. Still, surgical meshes are the preferential choice for restoring the physical integrity and equivalent components of musculofascial layers [1,8]. Nowadays, clinicians can choose from a variety of meshes, each with its own benefits and limitations [9]. However, an optimally performing such medical textile is yet to be found [10], and increasing research interest has been noted in improving polymer architecture, biocompatibility, operative handling, and cost of these reinforcement structures [8]. 

In this context, this review takes a comprehensive path to abdominal hernias, starting with a brief presentation of the pathology and general repair strategies, followed by a concise overview of currently available abdominal meshes and an extensive discussion on emerging scaffolds for hernia repair. Specifically, there are described the novelties in lightweight materials, meshes with improved attachment, antimicrobial fabrics, composite and hybrid textiles, and performant mesh designs. Additionally, recently completed clinical trials in the field are thoroughly detailed. 

Thus, despite the existence of several reviews describing mesh products [1,11,12,13,14,15], this paper brings a new perspective on the topic, updating the literature with the newest advancements. Broadly elaborating on the recently reported solutions for hernia repair, the present review aims to expose the state-of-the-art abdominal meshes, aiming to serve as an inception point for further research in the field.

## 2. Abdominal Wall Hernias—Pathology and Repair Strategies

“Hernia” is a term assigned to the abnormal protrusion of an organ or part of an organ outside its normally encased cavity. Hernias are most often encountered in the abdomen, being caused by the loss of continuity of the fasciae and/or muscles. They occur when the abdominal muscles are weakened, opened, or leaked, which produces a loss of intraabdominal pressure and outward protrusion of the abdominal structures [1,2,5,16]. The structural integrity of the abdominal wall can be disrupted due to multiple factors, including age, gender, genetic susceptibility, anatomic variations, obesity, smoking, constant heavy lifting, pregnancy, traumas, and postoperative complications of prior surgeries [1,13,16]. From a biological perspective, hernia occurrence has been linked to several molecular and cellular mechanisms, including abnormal extracellular matrix (ECM) metabolism (especially collagen metabolism), irregularities in growth factors and nutrition, and alterations in cell phenotypes. However, further thorough research is needed to better understand the specific cell-cell interactions and gene expression landscapes of abdominal hernia [1]. 

**Figure 1:**
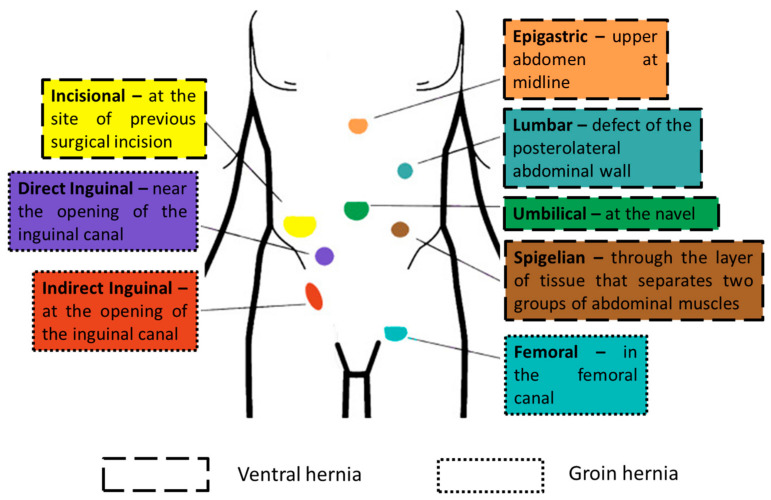
Location of different types of hernia. Adapted from an open-access source [13].

According to the World Society of Emergency Surgery (WSES), two main categories of abdominal wall hernias can be distinguished depending on their anatomical site: groin hernias and ventral hernias (Figure 1). The first category designates the hernias occurring at the bottom half of the body, including indirect inguinal, direct inguinal, and femoral hernias, whereas ventral hernias comprise several other types of disruptions, such as umbilical, epigastric, Spigelian, lumbar, and incisional hernias [13,17].

Abdominal wall herniation can cause discomfort or pain in the affected area, reduce mobility, and limit the patient’s daily activity. Moreover, it can negatively impact function, enhance size, cosmetically deform the abdomen, and compress abdominal contents, thus decreasing the quality of life of affected patients [5,13,16,18]. Therefore, hernias require careful monitoring and timely intervention to restore abdominal wall integrity and prevent further complications.

**Figure 2:**
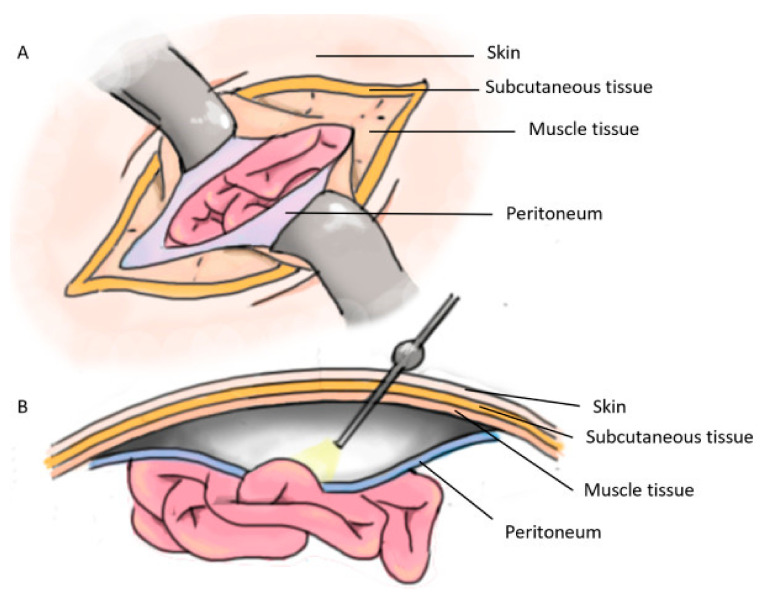
(**A**) Open repair surgery; (**B**) Laparoscopic surgery. Reprinted from an open-access source [13].

Therapeutic approaches depend on the size and severity of the hernias. Usually, if the hernia is not life-threatening, it is considered a safe strategy to only observe and monitor its evolution over time without intervening. Nonetheless, if the hernia is severe, surgical repair is imposed, and several operative procedures can be chosen. The most commonly employed option is open repair surgery (i.e., Lichtenstein procedure), in which the surgeon closes the hole through different fixation methods by making an incision above the defect (Figure 2A). A less invasive alternative is laparoscopic surgery (Figure 2B), a less often involved technique, generally utilized but not limited to hernias that reoccur. Both open repair and laparoscopic procedures can be performed on all types of hernias; the choice between them depends on the surgeons’ comfort and the patient’s decision. Laparoscopic approaches tend to be preferred, given their association with less pain and shorter hospitalization time, yet they require general anesthesia. On the other hand, open repair surgery results in a longer hospitalization period and a higher risk of infectious complications, but it only necessitates local anesthesia and is also easier to perform the procedure for a general surgeon [13,19,20,21].

**Figure 3:**
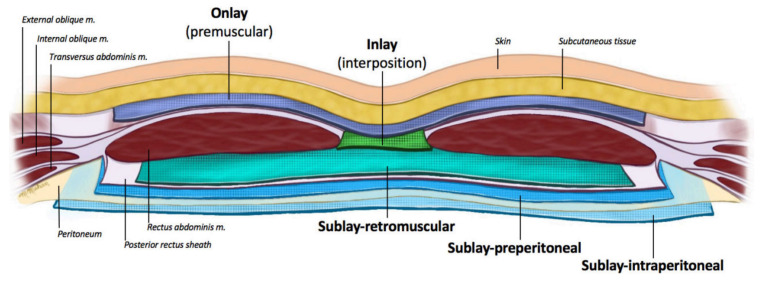
Schematic representation of the abdominal wall demonstrating mesh planes. Reprinted from an open-access source [23].

Even though each of these surgical approaches has its own benefits and limitations, the introduction of a mesh is considered a disruptive development in hernia repair [13]. Until the 1950s, simple sutures were used to close the abdominal wall, the surgeons opting for silk, silver, and polymeric sutures. However, such repair strategies were associated with high recurrence rates, suture rupture, and ischemia in treated patients [1,4]. With the introduction of the first polyethylene mesh in 1958, the field of hernia repair has faced tremendous progress, with a wide range of mesh products being developed since then [8]. Within the last years, mesh use has become a standard approach in hernia surgery, as these nettings provide mechanical support and a structure for tissue to “scar” into, consequentially reducing recurrence risks [4,22]. A mesh can be inserted in different anatomic sites to reinforce and stabilize the abdominal wall. The anatomic location of the implant is recognized to interfere with mesh integration within native tissues, the tensile strength of the repair, and the immune reaction at the interface between the implant and tissues. The optimal placement of these medical textiles is still debated among clinicians, yet the most prevalent choices include onlay, inlay, sublay-retromuscular, sublay-preperitoneal, and sublay-intraperitoneal (Figure 3).

## 3. Currently Available Abdominal Meshes

Hernia repair is favored by interdisciplinary technological advances, as its performant management benefits from developments in bioprosthetic devices, materials, surgical techniques, and combined approaches [24]. Concerning mesh options, a wide range of biomedical textiles are clinically available, each with advantages and disadvantages depending on placement position and usage circumstances [9]. In the following subsections, currently available meshes are briefly presented depending on their manufacturing materials, being divided into synthetic, biological, and composite meshes.

### 3.1. Synthetic Meshes

Synthetic meshes are generally viewed as the best option for abdominal wall-defect repair, demonstrating their clinical efficacy in many cases over a long time of utilization in hernia repair [16,25]. Polymeric meshes are considered advantageous due to their adequate elasticity and tensile strength that endow the textiles with the capacity to withstand intrabdominal wall pressures and prevent re-herniation. These porous architectures knitted from polymer fibers are also relatively inexpensive, being a cost-effective solution for hernia repair [4,16,25]. Numerous synthetic meshes are available on the market, a series of clinically used such textiles being presented in Table 1.

Depending on the manufacturing polymer, synthetic meshes can be either permanent (non-resorbable) or absorbable (resorbable) [16,26]. Non-resorbable meshes are durable textiles that are preserved as such indefinitely in the body. These meshes are a common choice for hernia repair. The fabricating materials mainly include polypropylene, polyester, polytetrafluoroethylene (PTFE), and expanded PTFE, but polyvinylidene fluoride and polyurethane are also valid options [16,22].

On the other hand, resorbable meshes are made of degradable materials that remain intact only for a definite period, as required by the wound (i.e., short-term: days-weeks, mid-term: weeks-months, or long-term: months-years). Such meshes can be obtained from biodegradable polymers, such as poly-4-hydroxybutyrate, polyglactin, polylactic acid, polyglycolic acid, polycaprolactone, and polyvinyl alcohol [16].

Some materials may degrade too soon, not ensuring proper tissue strength from cellular remodeling [25]. In contrast, non-resorbable polymers have been linked with more frequent foreign body reactions and adhesion. In this context, for the development of newer meshes, the combination of absorbable and non-absorbable polymers has been considered [22].

Despite their common utilization in practice, synthetic meshes are not always suitable. Synthetic meshes are inadequate for hernia repairs involving an open abdomen or in contaminated/infected fields because of their increased risks of adhesions, chronic sepsis, erosion, and subsequent enteric fistulation [25]. Permanent polymeric textiles are prone to postoperative infective complications and may require mesh removal [26].

**Table 1:** Examples of synthetic meshes currently on the market. Created based on information from [23,27,28].

Material	Commercial Name	Manufacturer
Polypropylene	Prolene	Boston Scientific (Marlborough, MA, USA)
	Marlex	Bard Davol (Warwick, RI, USA)
	Parietene	Covidien-Medtronic (Fridley, MN, USA)
	Surgipro	Covidien-Medtronic
	ProLite	Pierson Surgical (North Bradley, Trowbridge, UK)
Polyethylene terephthalate polyester	Dacron	DuPont (Wilimington, DE, USA)
	Mersilene	Ethicon (Johnson&Johnson) (Bridgewater, NJ, USA)
Polytetrafluoroethylene (PTFE)	Teflon	DuPont
Expanded polytetrafluoroethylene (ePTFE)	Gore-Tex	W.L. Gore and Associates (Newark, DE, USA)
Polyglycolic acid	Dexon	American Cyanamid (Bridgewater, NJ, USA)
Poly-4-hydroxybutyrate	Phasix	Bard Davol
Bioengineered silk	Seri	Sofregen Medical (Framingham, MA, USA)

### 3.2. Biological Meshes

Biological meshes are a feasible alternative to synthetic materials, being especially of use in infected fields within complex abdominal wall hernia repairs [4,25]. Biological materials exhibit an inherently lower inflammatory response and resilience, thus making them an attractive option for high-risk patients [4]. They can be derived from human (allograft) or animal (xenograft) tissues to isolate the extracellular matrix (ECM). ECM is the main component of biological meshes responsible for their structural, mechanical, and biochemical functions. The role of ECM is given by its rich collagen I content and the presence of signaling molecules that create a beneficial environment for wound healing and regeneration, allowing neovascularization and cellular repopulation through native fibroblasts infiltration [4,25,26,29].

Biological materials, such as the human dermis, porcine small intestine submucosa, porcine dermis, bovine dermis, and bovine pericardium, are processed to eliminate cells and DNA of the source to generate immunologically inert matrices. These acellular scaffolds may also be further crosslinked to avoid collagen degradation by blocking collagenase-binding sites while preserving the integrity of the material for a more extended period with slower incorporation into the adjacent tissue [26].

Despite their advantages, biological meshes are rather high-priced. Hence, their use is avoided in simple, clean hernia repair cases. Moreover, they are prone to stretching over time due to elastin protein retention [4,25]. Nonetheless, when the context calls, several biological meshes can be employed in clinical practice, given the market availability of human, porcine, and bovine-derived implantable scaffolds (Table 2).

### 3.3. Composite Meshes

Composite meshes started being employed in hernia repair as a method of circumventing the downsides of single-material scaffolds. A composite mesh can combine the benefits of two synthetic materials or one synthetic and one natural material toward achieving good integration within the host tissue while providing adequate mesothelialization at the peritoneal level. Consequently, complications generated by the implantation of a reticular material can be reduced, including adhesions, mesh migration, or intestinal fistula [5].

Composite meshes can retain the mechanical properties of classic non-absorbable polymers like polypropylene and polyethylene terephthalate while mitigating their potential complications by the addition of an absorbable netting layer [1]. Another exploited possibility is the fabrication of meshes with a synthetic layer facing the dermis (for ensuring the required mechanical strength and stimulating collagen accumulation and ingrowth) and a naturally degradable biomaterial layer facing the peritoneum (for preventing visceral adhesion) [17].

Considering these appealing features of composite meshes, several such products have been introduced on the market (Table 3).

### 3.4. Limitations of Currently Used Meshes

Clinicians have faced numerous difficulties in treating hernia patients because of the increasing variety of novel non-infectious and infectious complications associated with the widespread use of meshes. Encountered issues after mesh implantation include inflammation, wound healing problems, postoperative and chronic pain, seromas, adhesions, migration of the mesh, and implant rejection [13,22,40]. Other negative effects that can be triggered by an inappropriate mesh choice count fibrosis and calcification [22].

Despite not being correlated with high rates of surgical site infections, meshes are still perceived as foreign bodies by the host potentiating the generation of inflammatory responses, and, if an infection occurs in the region, it may further escalate to abdominal wall damage, increasing postsurgical pain and raising the recurrence risk [40]. Concerning infection risk, it was noted to also depend on underlying co-morbidities, patients with diabetes, immunosuppression, obesity, or smoking habit being more exposed [22]. Thus, special considerations should be taken when choosing appropriate meshes for these categories of patients.

To summarize the advantages and disadvantages of each of the discussed mesh categories, Table 4 was included.

## 4. Emerging Solutions for Performant Abdominal Meshes

**Figure 4:**
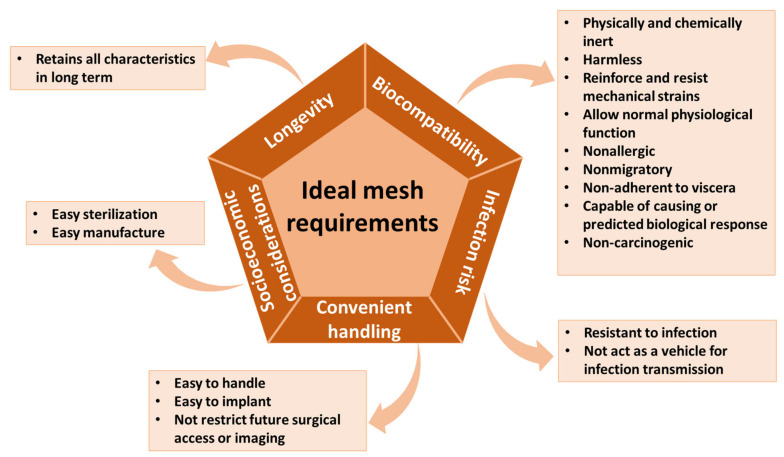
The main requirements of an “ideal mesh”. Created based on information from [10].

Despite the wide range of currently available mesh options, there is still room for improvement toward optimizing hernia repair management. Given that all existent material types present certain drawbacks, recent research aimed at creating an “ideal mesh” that would satisfy specific requirements concerning biocompatibility, infection risk, handling, longevity, and socio-economic considerations (Figure 4).

No ideal mesh has been developed yet. However, studies revealed that fulfilling mesh requirements depends on the choice of material, design, insertion technique, and mesh position with respect to the abdominal wall. A good mesh implies the fabrication from a strong, biologically inert, noncarcinogenic, and infection-resistant material that will generate a negligible foreign body reaction with no pathologic fibrosis [8,10,28]. The mechanical and biological properties of meshes are also related to textile type (i.e., woven or knitted), fiber type (i.e., mono- or multifilament), and pore size [22]. Pore size, in particular, may influence adhesions following intraabdominal placement, tissue integration, active surface area, elasticity, and memory [8]. Moreover, an ideal mesh should be able to remodel or regenerate into tissue similar to native fascia. In this respect, an ideal solution would be a polymeric scaffold incorporated with signaling molecules that can stimulate host immune cells and fibroblasts to regenerate human fascia [4].

Despite not finding an ideal mesh yet, several promising research directions comprise developing lightweight materials, improving mesh attachment, fabricating antimicrobial implantable textiles, formulating innovative composite and hybrid materials, and creating novel mesh designs. The following subsections describe these possibilities in more detail.

### 4.1. Lightweight Materials

“Lightweight” generally indicates a larger pore size that is reflected in a smaller surface area. Given that lightweight meshes present a reduced amount of material compared to their heavyweight counterparts, they are supposed to produce diminished foreign body reaction and fibrosis. It is also assumed that lightweight biomedical textiles are more flexible, exhibiting improved physical properties and allowing a better activity profile after surgery [8].

Several recent literature studies have demonstrated the superiority of lightweight meshes (LWM). For instance, Sidharta et al. [6] have reported lightweight textiles to be a better choice for reducing early postoperative pain from herniorrhaphy using the Lichtenstein technique in elderly men than heavyweight meshes (HWM). Lata et al. [41] have also compared HVM and LWM in Lichtenstein repair. The researchers noted more frequent chronic pain and a considerably higher foreign body sensation in the HWM group. In addition, HWM receivers complained of stiffness around the incisional site, while the patients treated with a LWM did not accuse of such discomfort. The benefits of LWM are also reflected in early mobility and an early return to work in receiving subjects.

The advantages of LWM have also been evaluated in other procedures. The study of Ahmed Abd El A and colleagues [42] has proven lower postoperative pain on the first postoperative day and after 1 week and earlier time to return to routine daily activities for LWM than for HWM when used as reinforcements in the laparoscopic transabdominal preperitoneal repair of inguinal hernia. However, a longer operative time was reported for LWM, while there was no considerable difference between the two groups in terms of chronic pain, postoperative complications, and recurrence after 6 months of follow-up. In contrast, RezK et al. [43] have compared LWM and HWM for ventral hernia repair, obtaining better clinical outcomes for the LWM group. Specifically, LWM use reduced chronic pain and foreign body sensation, less frequent complications (i.e., seroma and infection), and lower recurrence rates. Nonetheless, the cost-benefits of using LWM are still considered an important obstacle.

Thus, LWM represent superior alternatives to HWM for hernia repair as long as they are made from affordable materials. Moreover, longer-term studies employing larger patient cohorts are expected to provide clarifying information concerning durable LWM efficacy.

### 4.2. Materials with Improved Attachment

Most meshes currently employed in clinical settings have been linked to adhesion and chronic pain issues. If mesh attachment to the abdominal wall were improved, these biomedical devices would allow a faster and easier implant, leading to shorter operating times [8,44]. One way of enhancing mesh fixation to the host tissue is to manufacture self-fixation textile that anchors themselves through grips or adhesives, preventing unnecessary trauma from sutures or tacks usage [5].

For instance, Ben Yehuda et al. [45] have proposed a bio-adhesive-based self-fixation mesh (LifeMesh™) as an alternative to standard tack fixation. Their study revealed that, 28 days after insertion in a full-thickness abdominal wall defect of pig animal models, the bio-adhesive layered was substantially degraded, permitting tissue growth to become the new main fixation mode. Thus, LifeMesh implantation resulted in excellent incorporation into the abdominal wall, superior fixation strength, and low adhesion score, demonstrating better outcomes than conventional alternatives.

More recently, Harman and colleagues [46] have manufactured an innovative bio-adhesive-polypropylene mesh system that combines a bifunctional poloxamine hydrogel adhesive and a poly-glycidyl methacrylate (PGMA) layer grafted with human serum albumin. The newly developed system offered significantly improved adhesive strength compared to polypropylene mesh fixed with fibrin sealant. The bio-adhesive disintegrated, and 42 days after implantation in a rabbit model, tissue integration within mesh pores was observed, thus producing enough strength to withstand the physiological forces expected in hernia repair applications.

Another method to improve mesh attachment is introducing cellular components in the textile structure [44]. In this respect, Dong et al. [47] have created a composite electrospun scaffold made of a thermoresponsive hydrogel (i.e., poly (N-isopropyl acrylamide)-block-poly (ethylene glycol)) and a biodegradable polymer (i.e., polylactic acid) and seeded it with rat adipose-derived stem cells. The proposed alternative provided a highly biocompatible three-dimensional fibrous matrix with satisfactory mechanical strength and desirable biological properties. in more detail, the cell-seeded composite mesh could simulate the native ECM, accelerate cell adhesion and proliferation, improve defect repair and regeneration, and promote early vascularization.

On a different note, Lesage et al. [48] have seeded mesenchymal stem cells derived from amniotic fluid on electrospun polylactic acid matrices. The polymeric scaffold supported cell adherence and proliferation as at the 14 days benchmark, the meshes were well penetrated by inflammatory cells, new blood vessels, and collagen fibers. When implanted in rat models, stem cell addition to polymeric scaffolds was reported to modulate the host response after subcutaneous placement, with a similar macrophage profile compared to the control.

### 4.3. Antimicrobial Materials

To reduce the risk of infection, antimicrobial meshes can be employed in abdominal hernia repair. The two main possibilities of endowing the textiles with antimicrobial properties comprise placing an additional material layer in front of the mesh to slowly release an antimicrobial agent locally or embedding antimicrobials within the existing netting. Utilizing such antimicrobial meshes can potentially prevent bacterial adhesion and colonization, subsequently diminishing postoperative infection rates [22,49,50].

Several recent studies have focused on developing abdominal meshes with antimicrobial effects. For instance, Dydak et al. [51] have applied a layer of bacterial cellulose combined with gentamicin antibiotic on several types of polypropylene-based meshes. The researchers obtained encouraging results, the modified meshes showing enhanced bacterial growth inhibitory activity compared to non-coated textiles, thus hindering infection occurrence while preserving a high level of biocompatibility toward fibroblast cells.

Pérez-Köhler et al. [52] have also tackled the approach of applying an antibacterial coating to polypropylene meshes. The authors covered the synthetic meshes with a carboxymethylcellulose gel loaded with rifampicin. When tested in a preclinical model of Staphylococcus aureus and *S. epidermidis* infection in rabbits, the coated implants exhibited full bacterial clearance and optimal tissue integration, while bloodstream antibiotic levels remained undetectable. The same research group [53] has also investigated the in situ application of a rifampicin-loaded thermo-responsive hydrogel formulation onto implanted polypropylene meshes. Specifically, the antimicrobial formulation becomes a biodegradable gel at the implant site as it reaches body temperature, thus permitting an optimal coating of the mesh and surrounding tissues. The scientists reported a strong antibacterial activity lasting for 5 days without displaying any cytotoxicity signs. Thus, it was concluded that the designed gel is a potential complementary tool to abdominal meshes for preventing implant infections and improving tissue integration.

In recent years, nanotechnology has also gained ground for antimicrobial applications, the properties of nano-sized materials being appealing for creating numerous formulations effective in preventing and fighting against various infections [49,54,55,56,57,58]. Thus, it is no surprise that nanomaterials also became attractive for enhancing infection-resistant features of abdominal meshes. For example, Afewerki and colleagues [59] have created multifunctional bactericidal nanofibers with optimal properties for hernia repair. The authors engineered a blend of polycaprolactone methacrylated nanofibers and gelatin (denatured collagen) methacryloyl that has proven bactericidal activity, low inflammatory response, and good biodegradation. Moreover, their fibers displayed tunable mechanical properties with enhanced hydrophilicity and biological performance, being promising candidates for abdominal meshes capable of biointegration, blood vessel formation, and tissue ingrowth.

Differently, Liu et al. [60] have constructed a polycaprolactone/silk fibroin mesh integrated with amoxicillin-incorporating multi-walled carbon nanotubes. This complex nanofibrous architecture presented biocompatibility, mechanical properties similar to the abdominal wall, undeformed structure, and sustained release of the loaded antibiotic, further reflecting on efficient growth inhibition of *E. coli*.

Another appealing possibility is the incorporation of inherently antimicrobial nanoparticles within implantable textiles. Metal and metal oxide nanoparticles (e.g., silver, gold, copper, iron oxide, zinc oxide, titanium oxide, and magnesium oxide) are particularly recognized for their effectiveness against various pathogens, including antibiotic-resistant strains [49,55,56,61,62]. Hence, these nanomaterials have also attracted interest in the field of abdominal meshes, with several metal-based nanoparticles already being investigated for developing antimicrobial scaffolds useful in hernia repair [63,64,65,66,67].

### 4.4. Other Innovative Materials

In the effort to provide better alternatives to existent abdominal meshes, recent research has also produced a series of various other composite and hybrid materials that do not fall under the above-listed categories. One such example is the mesh formulation proposed by Li and colleagues [68], who have blended poly (l-lactide-co-caprolactone) with porcine fibrinogen in various ratios. The optimal physicomechanical features were reported for the 4 to 1 synthetic to biologic material, leading to desirable shrinkage rate, mechanical strength, porosity, and super-hydrophilic properties. Such characteristics are further reflected in an equilibrium between material degradation and host tissue growth, enabling proper tissue remodeling and reconstruction.

Alternatively, Mori da Cunha et al. [69] have developed a hydrogen-bonded supramolecular polymer made of ureidopyrimidinone moieties incorporated into a polycarbonate base. Compared to standard polypropylene meshes, the new composite displayed slightly better behavior, but the results were still suboptimal imposing additional material optimization studies.

A different innovative strategy is suggested by Liu et al. [70], who have combined polycaprolactone, silk fibroin, and decellularized human amniotic membrane. The hybrid material offered an adequate microenvironment for cell proliferation and neovascularization while provoking a weaker inflammatory response and foreign body reaction than polymer alone meshes. Another recent study investigated polycaprolactone-containing composites for the repair of abdominal wall defects. Liu and colleagues [71] have fabricated a double-layer structured nanofiber membrane using polycaprolactone, graphene oxide, and chitosan and loaded it with N-acetylcysteine. The as-designed mesh displayed excellent mechanical strength, biocompatibility, and collagen deposition, exhibiting good anti-hernia and anti-adhesion effects. One more adhesion-free composite material was reported by Chalony et al. [72], who fabricated a biocompatible non-woven material from poly (ethyl-2) cyanoacrylate reinforced by polyurethane. The composite fibrous architecture demonstrated adequate mechanical properties for repairing the viscera layer if used as intraperitoneal hernia mesh implants.

Otherwise, Wang et al. [73] have fabricated a poly-L-lactic acid scaffold grafted with a basic fibroblast growth factor (bFGF) on the surface that has the required mechanical properties and biocompatibility for hernia repair. The addition of bFGF improves the material’s hydrophilicity, its sustained release also regulating immune cytokines, inhibiting the inflammatory reaction, and enhancing collagen genes expression and protein secretion.

On a different note, Zhou et al. [74] have worked on a core-shell electrospun fibrous membrane with the center made of puerarin and the outer layer consisting of RGD surface modification. The core is responsible for long-term endogenous inflammation inhibition, whereas the RGD shell increases biocompatibility, promotes cell viability and adhesion, and ensures exogenous inflammation suppression. Moreover, testing on rat models revealed promising wound healing properties as the developed membrane stimulated collagen deposition, smooth muscle formation, and vascularization.

### 4.5. Novel Mesh Designs

Besides the fabrication material, mesh design was also noted to play an important role in the efficacy of hernia repair. Specifically, the shape and architecture of the reinforcement textiles can influence clinical outcomes.

A study by Minardi et al. [75] is worth mentioning in support of these statements. The researchers have prepared a type I collagen/elastin crosslinked blend (CollE) from which they fabricated flat sheets and porous scaffolds to serve as biomimetic meshes for ventral hernia repair. Both of the developed architectures have proven biomechanically adequate for sustaining immediate repair of hernia defects, ensuring tissue restoration in only 6 weeks, and promoting neovascularization. However, CollE scaffolds demonstrated more similar mechanical features to native tissues, inducing higher expression of genes related to de novo matrix deposition, angiogenesis, adipogenesis, and skeletal muscles, compared to CollE Sheets.

Furthermore, a recent article published by Amato et al. [76] described the successful use of a tentacle-shaped mesh that guarantees a fixation-free approach with a larger defect overlap in the repair of Spigelian hernias. The mesh composed of a central body with integrated radiating arms was tested in 54 patients. It was positioned in the preperitoneal sublay with the “tentacles” oriented across the abdominal musculature with a needle passer and cut short in the subcutaneous layer after fascia closure. This innovative design enabled an easy, fast, and safe fixation-free mesh placement with negligible complications, no recurrence, and greatly reduced pain.

In addition, through the careful choice of mesh fabrication method, the implantable textile’s properties can be influenced and optimized [77]. Currently, most mesh products are manufactured through the wrap-knitting process, with the morpho-structural characteristics being dictated by the direction of courses (rows) and wales (columns) of the fiber/yarn in relation to each other. This fabrication technique supposes curving fibers to form a meandering pattern, giving the device a more elastic and flexible architecture than woven materials and the ability to adjust to the movement of the human body. Nonetheless, this method also presents certain disadvantages, such as greater ultimate load values and no adaptation to the anisotropic mechanical behavior of normal human abdominal wall tissues [12,78]. As an alternative production method, electrospinning started being increasingly used, especially for generating fibers with diameters in the nano range. Electrospinning allows for easy manufacturing of polymeric scaffolds with large surface area-to-volume ratio and interconnected pores, being a versatile and cost-effective technique and offering enhanced control over fiber topography and orientation. However, electrospinning applicability is limited, resulting in products with poor mechanical properties and ineffectively controlled pore structures [79,80].

In this context, promising advances in mesh design have been envisaged by approaching additive manufacturing, a particularly favorable fabrication method being 3D printing. Such techniques grant the development of advanced, highly accurate, customizable patient-specific devices that conventional fabrication could not accomplish. Moreover, additive manufacturing is also convenient for rapid and facile surface modification of preexisting meshes [16,81,82]. With these advantages in mind, numerous researchers approached 3D printing to generate novel mesh designs with improved performance. Several studies reported on the production of promising 3D-printed meshes, including composite polylactic acid-acellular dermal matrix scaffolds [83], personalized polypropylene-polyvinyl alcohol meshes loaded with ciprofloxacin [84], drug-doped polycaprolactone meshes containing alginate and gentamicin [85], tailored meshes made of alginate and waterborne-polyurethane [86], and custom polycaprolactone constructs impregnated with iodinated, gadolinium, and barium contrast agents [87].

The so-called “4D printing” additive manufacturing technique also holds great promise for developing novel mesh designs. This method introduces time as the 4th dimension, aiming to recapitulate the dynamics of living tissues by smart thermopolymers that can change their shape in response to physicochemical or biochemical stimuli. Employing stimuli-responsive materials would allow the creation of performant meshes to progressively accustom and react to modifications in the host-tissue environment, improving tissue ingrowth and implant compliance [16]. To our knowledge, this technique has not been used for designing abdominal meshes yet. However, its emergence in fabricating other types of polymeric scaffolds for adaptive biomedical implantation [88,89,90,91,92,93] represents an inception point for future research oriented toward hernia repair.

One more emerging possibility for improving mesh design is the application of embroidery technology. In contrast to the warp-knitting method, embroidery permits the generation of personalized designs. Specifically, custom-made patterns can have the thread direction organized at nearly any angle with minimal realization effort and minor machine adjustments. While not yet employed for fabricating hernia meshes, the potential of embroidery has been envisaged for producing tissue-engineered scaffolds [78,79], holding promise for soon-generating implantable textiles with better-controlled designs.

### 4.6. Clinical Trials

In addition to the above-mentioned top-recent developments, numerous strategies have reached the clinical testing stages, indicating the increased interest in this field and the acute need to introduce into practice better-performing abdominal meshes. Searching the term “abdominal mesh” in relation to “hernia” (completed in the “condition or disease” field) on the ClinicalTrials.gov platform resulted in the retrieval of 418 studies. According to their status, there are 16 “not yet recruiting”, 62 “recruiting”, 8 “active, not recruiting”, 218 “completed”, 28 “terminated”, 10 “withdrawn”, and 76 “unknown” status clinical trials. In what concerns study phase, there have been identified 35 “phase 4”, 14 “phase 3”, 17 “phase 2”, 4 “phase 4”, 1 “early phase 1”, and 245 studies for which this criterion does not apply. 308 clinical trials are “interventional” and 110 are “observational”. 47 studies have publicly available results, whereas the remaining 371 studies are “without results”.

By restricting the search to “completed studies” and “with results”, a number of 38 clinical trials were retrieved from the platform and tabulated in Table 5.

Among the listed completed studies that have results, several clinical trials have been extensively discussed in a number of publications. For instance, NCT02451176 [94] was discussed in two articles [132,133], comparing the outcomes of biologic and synthetic meshes used for single-stage repair of clean-contaminated and contaminated ventral hernias. The study was conducted on 253 adult patients from December 2012 to April 2019 with a follow-up duration of 2 years. The results revealed a superior 2-year hernia recurrence risk for synthetic meshes implanted in patients with contaminated ventral hernias, whereas the price of biologic mesh was over 200 times that of synthetic mesh for these outcomes. However, both meshes demonstrated similar safety profiles, and the overall and hernia-related quality of life were similarly improved in both study groups. Treatment of contaminated ventral hernia repair was also assessed by NCT00617357 [115], yet from a different point of view. Researchers have investigated the influence of mesh placement, all involved patients receiving an intact, non-cross-linked, porcine, acellular dermal matrix (Strattice™, LifeCell, Branchburg, NJ, USA). According to associated publications [134,135], the Strattice mesh was placed in the retro-rectus position in 23 patients and in the intraperitoneal position in 26, leading to successful reconstruction in >70 of patients at the 2-year repair follow-up benchmark. Despite the larger hernia defects identified in the retro-rectus group, positioning the reinforcing netting in the retro-rectus compartment resulted in a similar recurrence rate to intraperitoneal mesh placement yielded a similar recurrence rate to intraperitoneal mesh placement. Nonetheless, the validity of these findings should be confirmed by evaluating longer-term results. Strattice mesh was the object of investigation of NCT00681291 [123] as well, being compared to a lightweight, large pore polypropylene mesh (UltraPro(TM), Ethicon, Somerville, NJ, USA). The study focused on comparing the safety and effectiveness of the two materials in a Lichtenstein inguinal hernia repair and evaluating postoperative complications, chronic pain, and re-herniation occurrence at 12 and 24 months [136]. However, results are yet to be elaborated and discussed in future publications.

Another evaluation was realized through study NCT00815698 [113], where two strategies were employed for open primary repair of uncomplicated inguinal hernia by the Lichtenstein technique. Scientists comparatively investigated a self-gripping mesh (Parietene Progrip^®^, Sofradim, Trevoux, France) and sutured mesh on 163 and 171 patients, respectively. The researchers did not observe any significant differences between the tested groups in terms of postoperative complications, rate of recurrent hernia within 1 year, or quality of life [137]. On a different note, study NCT01848184 [128] assessed the clinical outcomes two years after patients underwent open ventral primary hernia repair with the use of the Parietex™ Composite Ventral Patch. Mesh use was associated with a low recurrence rate, low postoperative and chronic pain, and high satisfaction ratings, rendering it an effective solution for small ventral hernia repair [138].

Alternatively, the NCT02720042 study [95] assessed the efficacy of biosynthetic mesh Phasix™ (Bard Davol, Warwick, RI, USA) over 24 months in grade 3 hernia adult patients. The implant placed in a sublay position was expected to provide the biocompatibility and resorbability of a biological mesh while preserving the mechanical strength of a synthetic mesh [139]. Unfortunately, the clinical trial results revealed no significant difference in the quality of life of treated patients nor a considerable pain reduction. Moreover, 10.7% of study subjects reported the lasting presence of mesh sensation in daily life 24 months after surgery [140]. The same mesh was employed throughout NCT01863030 clinical trial [85], used in 31 patients who underwent ventral and incisional hernia repair via a Rives-Stoppa approach with retro-rectus mesh placement. As reported by Plymale et al. [141], the biosynthetic mesh was found to be a feasible solution for the patient cohort, leading to an improved quality of life in treated patients 2 years after surgery. In addition, early hernia recurrence is lacking among the patient population. For more concluding results, Phasix^TM^ was also evaluated throughout a longer-term study (i.e., NCT01961687 [104]). 121 comorbid patients with class I wounds underwent ventral and incisional hernia repair with the named biosynthetic mesh. Among the 54 patients who completed the 60-month follow-up, ~22% exhibited recurrence, and ~10% presented surgical site infection. Along the entire cohort, patients reported decreased pain, 18 of 121 required reoperations, and 9 of 121 developed seroma requiring intervention. Thus, it was concluded that the use of this specific mesh is linked with infrequent complications on the long term and durable hernia repair outcomes [142].

Two publications have also been identified in relation to clinical trial NCT03074474 [121], the first one discussing its outcomes 12 months after intervention [143] and the second one addressing results observed 24 months after ventral hernia repair [144]. Specifically, the study evaluated the performance of a reinforced tissue matrix (OviTex^®^ 1S–TELA Bio Inc., Malvern, PA, USA) in 92 patients with high risk for surgical site recurrence, out of which 65 completed the 24-month follow-up. Despite the delicate cohort, the utilized mesh has been revealed as a viable option for ventral hernia repair, leading to low recurrence and intervention rates [143,144]. However, longer-term studies may be required before drawing definitive conclusions.

Studies have also analyzed meshes of different densities to establish the most appropriate choice for hernia repair. Clinical trial NCT03082391 [101] compared medium-weight with heavyweight polypropylene meshes, yet no significant clinical or patient-perceived difference was noted one year after using these nettings for open retro-muscular ventral hernia repair. Scientists concluded that long-term follow-up evaluations will elucidate potential variations between the durability and efficacy of these mesh materials [145]. Alternatively, ProGrip™ self-gripping lightweight polyester mesh and a standard polypropylene lightweight mesh fixed with sutures were compared in study NCT00827944 [107]. In this respect, adult men undergoing Lichtenstein repair for primary inguinal hernia were evaluated within the first 3 months and 1 year after surgery. Despite being well-tolerated and reducing early postoperative pain without increasing the risk of early recurrence, ProGrip™ did not reduce chronic pain [146]. Otherwise, study NCT01586741 [105] investigated retro-muscular inset of a lightweight polypropylene mesh versus dual closure of the anterior rectus fascia using Quill self-locking technology for abdominal rectus muscle diastasis. The researchers did not observe any difference in terms of early complications and perceived pain at the 3-month follow-up, both techniques being considered equally reliable concerning adverse outcomes during the early postoperative phase. However, the subject receiving the lightweight mesh experienced a better improvement in muscular strength [147].

Several studies also revolve around the use of anesthetics. For example, NCT02055053 [130] involved 70 patients that received unilateral laparoscopic total extraperitoneal (TEP) inguinal hernia repair, with control patients receiving 10 ml of 0.9% saline instilled into preperitoneal space while the treatment group received 10-ml 0.5% bupivacaine without epinephrine. However, there was noted no considerable difference in postoperative pain between the two groups [148]. In contrast, study NCT00626886 [127] evaluated the safety and efficacy of a drug-carrier mesh (i.e., XaraColl) that can deliver up to 200 mg of bupivacaine hydrochloride. XaraColl mesh was able to reduce the pain of receiving patients, these study subjects also taking significantly less opioid analgesia than placebo patients [149]. Differently, the patients involved in study NCT02007096 [119] underwent laparoscopic ventral hernia and were randomly assigned to get a transversus abdominis plane (TAP) block or placebo injection. As presented by Fields et al. [150], the TAP-receiving patients had significantly lower pain scores in the postanesthesia care unit and used less opioids than the control group at each time point assessed after 6 h postoperatively. Nonetheless, 24 h after intervention, there was no significant difference in pain scores between the two groups, concluding that TAP blocks are an effective short-term solution for diminishing postoperative opioid use and pain experienced by patients.

A few studies that compared laparoscopic and robotic hernia repair were explored in associated publications. Bilezikian et al. [151] discussed the results of NCT01825187 [109], in which two different lightweight polypropylene meshes were inserted through the two surgical strategies for treating inguinal hernias in 48 patients. It was observed that robotic mesh placement significantly increased insertion time regardless of mesh type, mainly attributed to the mechanics of robotic suturing and the associated learning curve. Similar findings were also reported by Petro et al. [152,153] based on the outcomes of study NCT03283982 [112]. In addition to increased operative time, the researchers also registered higher costs for the robotic approach due to additional operating room time. Concerning hernia repair outcomes, there was no significant difference in postoperative pain intensity after 1 year, length of stay and complication rates were similar, yet the quality-of-life scores favor the robotic alternative. In contrast, the recurrence rate is lower when employing the laparoscopic approach. Given that these results were obtained based on evaluating 33 laparoscopic repairs and 38 robotic repairs, further investigations should be performed on larger patient cohorts to verify their potential significance.

## 5. Conclusions and Future Perspectives

To summarize, mesh reinforcement has produced a disruptive shift in how hernias are treated worldwide, becoming an essential aspect of abdominal hernia repair. A wide range of materials and products have been adopted for the management of abdominal wall defects, each with its advantages and disadvantages. Nonetheless, searching for an ideal mesh represents an ongoing process, with better solutions being envisioned and researched in recent years. Hernia repair strategies may be upgraded by using lightweight meshes with improved attachment, mechanical properties, antimicrobial effects, and performant designs. Numerous clinical trials have recently investigated the use of innovative mesh products, some revealing promising results. Moreover, positive outcomes can be expected from undergoing studies as well, especially from those in advanced phases of testing.

To conclude, hernia mesh design has faced great advances in the last years, and the increasing research interest in the field has the potential to bring solutions from the laboratory to the market in the near future, improving the quality of life of millions of patients worldwide.

## Figures and Tables

**Table 2:** Examples of biological meshes currently on the market. Created based on information from [23,26,27].

Biological Source	Commercial Name	Manufacturer
Human dermis	Alloderm	LifeCell (Branchburg, NJ, USA)
	Allomax	Bard Davol
	Flex HD	Ethicon (Johnson&Johnson)
	Cortiva	RTI Surgical (Deerfield, IL, USA)
	Epiflex	DIZG (Berlin, Germany)
Porcine dermis	Strattice	LifeCell
	Permacol	Covidien-Medtronic
	Collamed	Bard Davol
	XenMatriX	Bard Davol
	XCM Biologic	Ethicon (Johnson&Johnson)
	Fortiva	RTI Surgical
	Cellis	Meccellis Biotech (La Rochelle, France)
Porcine intestine	FortaGen	Organogenesis (Canton, MA, USA)
	Biodesign/Surgisis	Cook Medical (Bloomington, IN, USA)
Bovine dermis	SurgiMend	TEI Biosciences (Princeton, NJ, USA)
Bovine pericardium	Veritas	Baxter (Deerfield, IL, USA)
	Tutomesh	RTI Surgical
	Periguard	Synovis Surgical Innovations (St. Paul, MN, USA)
	Tutopas	Mentor Corp (Irvine, CA, USA)

**Table 3:** Examples of composite meshes currently on the market.

Commercial Name	Manufacturer	Materials	Ref.
Gore Bio-A	W.L. Gore and Associates	Polyglycolic acid reinforced with trimethylene carbonate	[30]
Tigr Matrix	Novus Scientific (Uppsala, Sweden)	Knitted fibers of a copolymer of glycolide, lactide, and trimethylene carbonate and a copolymer of lactide and trimethylene carbonate	[31]
Parietex	Medtronic	3D monofilament polyester textile with hydrophilic absorbable collagen film	[32]
Parietene	Medtronic	Transparent macroporous polypropylene on one side and absorbable collagen film on the other side	[33]
Sepramesh	Bard Davol	Polypropylene mesh with a hydrogel safety coating	[34]
Composix	Bard Davol	Polypropylene mesh with a submicronic ePTFE barrier	[35]
DynaMesh-IPOM	DynaMesh (Aachen, Germany)	Dual-component structure made mainly of high-purity of polyvinylidene fluoride and a small proportion of polypropylene.	[36]
Proceed	Ethicon (Johnson&Johnson)	Polypropylene mesh with a layer of oxidized regenerated cellulose and polydioxanone suture polymer film	[37]
Vicryl	Ethicon (Johnson&Johnson)	Polypropylene-polyglactin 910 absorbable woven/knitted composite mesh	[38]
ProGrip	Medtronic	Macroporous, monofilament, polyester, or polypropylene mesh incorporating thousands of poly-lactic acid resorbable microgrips	[39]

**Table 4:** Overview of advantages and disadvantages of commercially available meshes for hernia repair. Created based on information from [13,17].

Mesh Type	Advantages	Disadvantages
**Synthetic**	Good mechanical strength Low cost	Prone to inflammation Stiffness Pain High rate of infection Formation of fistulae
**Biologic**	Mild inflammation Low formation of fistulae Reduced fibrosis	More expensive Lower mechanical strength
**Composite**	Low formation of fistulae	Various degrees of inflammation

**Table 5:** Summary of completed clinical trials.

ClinicalTrials.Gov Identifier	Official Title	Intervention/Treatment	Phase	Ref.
NCT02451176	A Prospective Randomized Trial of Biologic Mesh Versus Synthetic Mesh for the Repair of Complex Ventral Hernias	Device: Davol Bard Soft Mesh synthetic Device: LifeCell Strattice Reconstructive Tissue Biologic	Not Applicable	[94]
NCT02720042	A Post-Market, Prospective, Multi-Center, Single-Arm Clinical Investigation of Phasix™ Mesh for VHWG Grade 3 Midline Hernia Repair	Device: Phasix™ Mesh	Not Applicable	[95]
NCT01364233	A Prospective Outcome Study of Condensed Fenestrated PTFE Mesh (MotifMESH) in Non-sterile Abdominal Wall Defects	Device: MotifMESH	Not Applicable	[96]
NCT03247985	Randomized Prospective Single-Blinded Study of Totally Extra Peritoneal Inguinal Hernia Repair: Tacking Mesh Versus Self-fixating Mesh	Device: PROLENE Polypropylene Tacking Mesh Device: ProGrip Self-fixating Mesh	Not Applicable	[97]
NCT00960011	Randomized Study of Self Gripping Semi-resorbable Mesh (PROGRIP) With Polypropylene Mesh in Open Inguinal Hernia Repair—the 6 Years Result	Device: PROGRIP Device: POLYPROPYLENE	Not Applicable	[98]
NCT01117337	Comparing Non-fixation of Mesh to Mesh Fixation in Laparoscopic Total Extraperitoneal Inguinal Hernia Repair Under Spinal Anesthesia- A Randomized Controlled Trial	Procedure: Mesh Fixation	Phase 4	[99]
NCT01863030	A Prospective, Observational Study Utilizing Phasix™ Mesh During Ventral and Incisional Hernia Repair Surgery	Device: Phasix mesh implant	Observational study	[100]
NCT03082391	Long-term Results of Heavy Weight Versus Medium Weight Mesh in Ventral Hernia Repair	Device: Heavy weight Mesh Device: Medium weight Mesh	Not Applicable	[101]
NCT03450473	A Prospective Case Series Evaluating Surgimend Mp^®^ In Patients Undergoing Complex Abdominal Hernia Repair	Device: SurgiMend^®^ MP	Not Applicable	[102]
NCT02053168	A Prospective, Multicenter All Comers Study of a Novel Resorbable Mesh (Phasix Mesh) for Ventral or Incisional Hernia Repair	Device: Resorbable Mesh	Not Applicable	[103]
NCT01961687	A Prospective, Multi-Center Study of Phasix™ Mesh for Ventral or Incisional Hernia Repair	Device: Resorbable Mesh	Not Applicable	[104]
NCT01586741	A Prospective Randomized Study to Evaluate Two Different Surgical Methods for Treatment for Abdominal Wall Diastasis	Procedure: Quill suture application for repair or polypropylene mesh	Not Applicable	[105]
NCT02206828	Observational Registry Study for Symbotex™ Composite Mesh in Ventral Hernia Repair	-	Observational study	[106]
NCT00827944	ProGrip Mesh Repair Versus Lichtenstein Mesh Repair: a Comparative Randomized Study in Primary Inguinal Hernia	Device: Parietex Progrip Device: Low weight polypropylene mesh	Phase 4	[107]
NCT02500056	Single-centre Single-blinded Randomised Study Evaluating the Impact of Mesh Pore Size on Chronic Pain After Lichtenstein Hernioplasty	Device: Optilene LP mesh Device: Ultrapro mesh	Not Applicable	[108]
NCT01825187	Prospective Randomized Controlled Trial Comparing Resident Performance and Clinical Outcomes With Two Different Polypropylene Meshes for Laparoscopic Inguinal Hernias	Device: ULTRAPRO Mesh Device: 3DMAX Other: Evaluation	Not Applicable	[109]
NCT03495154	A Multi-center Post-market Single Arm Prospective Study of Parietene™ DS Composite Mesh in Subjects Undergoing Ventral Hernia Repair	Device: Parietene DS Composite Mesh	Not Applicable	[110]
NCT01325792	Prospective, Multicenter, Observational Study Evaluate Single-Staged Open Complex Ventral Incisional Hernia Repair Using a Biosynthetic Material for Midline Fascial Closure Reinforcement	Device: GORE^®^ BIO-A^®^ Tissue Reinforcement	Observational study	[111]
NCT03283982	Registry-Based, Prospective, Single-Blind, Randomized Controlled Trial: Robotic vs. Laparoscopic Ventral Hernia Repair With Intraperitoneal Onlay Mesh (IPOM)	Device: Robotic Ventral Hernia Repair with IPOM Device: Laparoscopic Ventral Hernia Repair with IPOM	Not Applicable	[112]
NCT00815698	Effect of Suture for Mesh Fixation in Lichtenstein Hernia Repair, a Prospective Controlled Randomized Trial	Procedure: suture Procedure: no suture	Not Applicable	[113]
NCT02457728	Mesh Fixation and Closure of Peritoneum Following Laparoscopic Hernia Repair Using N-butyl Cyanoacrylate	Device: LiquiBandFix8	Not Applicable	[114]
NCT00617357	A Multicenter, Prospective, Observational Evaluation of Repair of Infected or Contaminated Hernias (RICH) Using LTM	Device: LTM (Strattice Reconstructive Tissue Matrix)	Not Applicable	[115]
NCT00393887	Inguinal Hernia Study: A Double Blinded Randomized Prospective Study	Device: Biodesign IHM Device: Polypropylene mesh	Not Applicable	[116]
NCT02712398	A Prospective, Multi-center Trial of a Long-term Bio-Absorbable Mesh With Sepra Technology in Challenging Laparoscopic Ventral or Incisional Hernia Repair	Device: Phasix™ ST	Not Applicable	[117]
NCT00749268	Evaluation of Postoperative Pain Following Laparoscopic Hernia Repair: A Prospective, Randomized Comparison to Evaluate the Incidence of Postoperative Pain Associated With Absorbable Fixation (AbsorbaTack) vs Conventional Fixation (ProTack) Following Laparoscopic Hernia Repair	Device: AbsorbaTack Device: ProTack	Phase 4	[118]
NCT02007096	Transabdominal Plane (TAP) Blocks in Ventral Hernia Repair	Drug: Transabdominal Plane Block Drug: Non Transabdominal Plane Block	Phase 2	[119]
NCT01268514	ENHANCE: A Prospective Long-term EvaluatioN of the Use of Permacol™ Biological Implant in tHe Repair of Complex AbdomiNal Wall CasEs	-	Observational study	[120]
NCT03074474	A Prospective, Single Arm, Multi-center Study Evaluating the Short-term Clinical Outcomes of Ventral Hernias Treated With OviTex Reinforced Bioscaffold	Device: OviTex 1S Permanent	Not Applicable	[121]
NCT03750461	Stoma Closure and Reinforcement (SCAR) Trial—A Single Center Pilot Study of the Safety of a Mesh Reinforcement of Ileostomy Closure to Prevent Hernia Formation in Left Sided Colon and Rectal Cancer Patients	Device: Mesh Implantation	Not Applicable	[122]
NCT00681291	Prospective, Randomized, Controlled, Third-Party Blinded Multicenter Evaluation of Strattice/LTM in the Repair of Inguinal Hernias	Device: Inguinal hernia repair with Ultrapro Device: Inguinal hernia repair with Strattice	Phase 4	[123]
NCT01597128	Feasibility Study of The Use of FLEX HD^®^ Surgical Implant or STRATTICE^®^ Reconstructive Tissue Matrix in The Closure of Abdominal Wall Defects With Component Separation in Clean or Contaminated Cases	Device: Flex HD Device: Strattice	Not Applicable	[124]
NCT01070693	Randomized Clinical Trial of Lichtenstein Patch or Prolene Hernia System for Inguinal Hernia Repair	Procedure: Open mesh inguinal hernia repair Device: Prolene Hernia System Procedure: Lichtenstein technique	Not Applicable	[125]
NCT00866814	A Prospective, Single Arm, Multi-Center Study Of Open Ventral Hernia Repair Utilizing The Bard Ventrio Hernia Patch	Device: Bard Ventrio Hernia Patch	Observational study	[126]
NCT00626886	A Phase II, Randomized, Single Dose, Double-blind, Placebo-controlled Study to Investigate the Efficacy, Safety and Pharmacokinetic Profile of the CollaRx^®^ Bupivacaine Implant in Men After Open Mesh Herniorrhaphy	Drug: Bupivacaine Collagen Sponge Drug: placebo collagen sponge	Phase 2	[127]
NCT01848184	A Multicentre Prospective Study in Patients Undergoing Ventral Hernia Repair by Open Approach With Intraperitoneal Positioning Using Parietex™ Composite Ventral Patch—Panacea Study	Device: PARIETEX™ Composite Ventral Patch	Observational study	[128]
NCT01481376	Laparoscopic Transabdominal Preperitoneal (TAPP) Inguinal Hernia Repair With Self-fixating Parietex™ ProGrip™ Mesh: A Retrospective Study With 12 Month Follow-up	-	Observational study	[129]
NCT02055053	A Randomized, Double-blinded, Placebo-controlled Trial of the Effects of Infusing Local Anesthesia on Postoperative Pain During Laparoscopic Inguinal Hernia Repair	Drug: 0.5% Bupivicaine	Phase 4	[130]
NCT00528970	A Multicenter, Randomized, Double-Blind, Placebo-Controlled, Parallel-Group Study of Intravenous Methylnaltrexone (MOA-728) for the Treatment of Post Operative Ileus After Ventral Hernia Repair	Drug: MOA-728 Drug: Placebo	Phase 3	[131]
